# Advancing Global Health Equity: The Role of the Liberal Arts in Health Professional Education

**DOI:** 10.1007/s10912-023-09827-9

**Published:** 2023-12-16

**Authors:** Abebe Bekele, Denis Regnier, Tomlin Paul, Tsion Yohannes Waka, Elizabeth H. Bradley

**Affiliations:** 1 University of Global Health Equity, Kigali, Rwanda; 2 Vassar College, Poughkeepsie, NY USA

**Keywords:** Medical education, Global health, Liberal arts

## Abstract

Much innovation has taken place in the development of medical schools and licensure exam processes across the African continent. Still, little attention has been paid to education that enables the multidisciplinary, critical thinking needed to understand and help shape the larger social systems in which health care is delivered. Although more than half of medical schools in Canada, the United Kingdom, and the United States offer at least one medical humanities course, this is less common in Africa. We report on the “liberal arts approach” to medical curricula undertaken by the University of Global Health Equity beginning in 2019. The first six-month semester of the curriculum, called Foundations in Social Medicine, includes courses in critical thinking and communication, African history and global political economy, medical anthropology and social medicine, psychology and health, gender and social justice, information technology and health, and community-based training. Additionally, an inquiry-based pedagogy with relatively small classes is featured within an overall institutional culture that emphasizes health equity. We identify key competencies for physicians interested in pursuing global health equity and how such competencies relate to liberal arts integration into the African medical school curriculum and pedagogical approach. We conclude with a call for a research agenda that can better evaluate the impact of such innovations on physicians’ education and subsequent practices.

## Introduction

The number of medical schools on the African continent has grown substantially in the last 50 years, from about 50 medical schools in 1980 to more than 300 by 2022 (Monekosso 2014; Brimmo et al. 2022). The vast majority of African medical schools were shaped by the European model of undergraduate medical education, conferring an MBBS or MD degree with six to seven years of classroom-, hospital-, and laboratory-based experiential learning after high school (Monekosso 2014). In addition to the efforts of African governments, the Medical Education Partnership Initiative (MEPI) efforts beginning in 2001, funded largely through the United States President’s Emergency Plan for AIDS Relief (PEPFAR), have made important investments to support African medical schools’ expansion—not only in their clinical missions but also to develop research centers for improving the evidence base of medicine in Africa. Despite the increasing supply of physicians in Africa, extreme global health inequities persist (GBD 2015 Healthcare Access and Quality Collaborators 2017). In order to tackle these persistent inequities, new models of medical education are needed. The late Paul Farmer and others have suggested that we need a “new equity plan,” supported by physicians who are educated in social medicine and who integrate, rather than balkanize, the fields of public health and medicine (Farmer 2019). With such an orientation, physicians might be better positioned to accelerate health system improvements and advocate for greater health equity globally.

Much innovation has taken place in the development of medical schools across the African continent (Greysen et al. 2011; Mullan et al. 2011). New areas of focus have supplemented the traditional medical curriculum, including the use of integrated curricula, interprofessional education (to improve coordination among varying health professionals), community-based education to ensure medical students see firsthand the influence of the social determinants of health, and added use of technology (Greysen et al. 2011; Mullan et al. 2011). Nevertheless, less attention has been paid to education that enables multidisciplinary approaches and critical thinking that may be useful in influencing the larger social systems in which medicine is practiced and enhancing global health equity.

## The Liberal Arts Approach to Education

Liberal arts education, rooted in the Ancient Greek inquiry-based mode of education in the context of a democracy of free people, is designed to “liberate the mind,” encouraging students to ask questions, dialogue with each other and the instructor, and explore non-obvious approaches to diagnosis and problem-solving. A liberal arts approach encourages lifelong learners to 1) become familiar with multiple perspectives, 2) employ critical thinking, 3) use primary sources and be embedded in the community, 4) question the status quo and apply creative problem-solving, and 5) take an expansive view of social forces that shape power relations, human experience, and action. Previous models of integrating both the humanities and the arts into medical school curricula have been described (Macneill 2011; Moniz et al. 2021). However, these models focus on particular courses and experiences as supplementing the existing medical curriculum. In contrast, the liberal arts approach we describe includes not only course content across the humanities, arts, and social sciences but also attention to both the pedagogy and classroom culture in which all medical school courses are provided. Within the context of medicine, the liberal arts approach can set an essential foundation for medical students and physicians not only to identify the root causes of health inequities but also to convene the broader stakeholders (i.e., politicians, businesspeople, researchers) who play a role in creating and potentially remedying such inequities.

Despite the promise of a liberal arts approach, several challenges lie ahead in integrating the liberal arts into medical school curricula in Africa. African program developers must balance the universals of human experience with the particularities of the African context (Kaya and Seleti 2013; Eichbaum et al. 2019). In the post-colonial contexts of Africa, humanistic learnings and truisms often associated with Western literature and philosophy may work against or eclipse indigenous understandings of humanity, limiting rather than opening students’ minds to new ideas (Kaya and Seleti 2013). Additionally, in resource-limited settings (in terms of faculty and teaching materials), it may be challenging for medical schools and physicians (Eichbaum et al. 2019) to protect time for a broader approach to medicine that includes the humanities and social sciences. Overcrowding of the existing curriculum in a fixed amount of time—known as curriculum hypertrophy (Abrahamson 1978)—is also a risk. Moreover, medical schools may fall into the “content trap” (Eichbaum et al. 2019), presuming that specific courses are needed to impart humanistic competencies—rather than recognizing that curiosity, empathy, reflection, and imagination can be developed through a variety of curricular and pedagogical approaches, not merely a set of pre-defined classes.

## Rwanda: The University of Global Health Equity and the Liberal Arts

The University of Global Health Equity (UGHE) was founded in 2015 in Butaro, northern Rwanda, with a grand vision of training a new generation of health care professionals. UGHE is owned by Partners in Health (PIH) and is focused on integrating social medicine into the training of physicians and other health professionals. Inspired by Dr. Paul Farmer’s commitment to health equity and social justice, as well as the role of social medicine in addressing not just medical problems but also the larger structural problems of society in which medical problems emerge, UGHE opened its doors to dual degree students pursuing medicine and a Masters in Global Health Delivery (MBBS and MGHD) in 2019 (Drobac and Morse 2016). UGHE was settled in a rural area of Rwanda to recognize the importance of and to prepare health professionals to address the multi-faceted social determinants of health that are characteristic of rural settings throughout the continent. To this end, UGHE’s departments and centers focus on academic research, community engagement, and organizational development practices. To broaden its focus on promoting global health equity, the medical school curriculum has been shaped to include community-based training and research, interprofessional education, and a One Health approach that recognizes the interrelationships between people, animals, plants, and the shared environment.

The liberal arts approach has been critical to the medical school curriculum at UGHE. The curriculum (delivered in 375 classroom hours) includes courses in African history and political economy, psychology, gender equity and social justice, medical anthropology, critical thinking and scientific reasoning, technology and health, and writing and communication. The vast majority of these courses are provided in collaboration with faculty from Vassar College and Harvard University during the first six months of the six-and-a-half-year medical degree program, allowing for early exposure to these concepts. It is anticipated that these courses will broaden perspectives on determinants of health and deepen the understanding of medical situations physicians will encounter in their training and beyond. Students are also supported through mentorship activities that enable them to have one-to-one learning with faculty.

In addition to these specific courses, UGHE is experimenting with innovative pedagogical approaches and efforts to build an open, inquiry- and discussion-based classroom culture consistent with a liberal arts education. Concretely, this means course instructors having strong facilitation skills, the ability to create psychologically safe (Edmondson 1999) classroom spaces for intellectual debate and discussion, and a commitment to using diverse primary sources to challenge students to think critically and carefully about evidence and inference. Group work, case studies, oral presentations, use of media such as podcasts and blogs, and engagement of the arts all contribute to more innovative pedagogy and inclusive, engaged classroom and school culture—particularly if implemented with the institutional goals of equity and inclusion across students, staff, and faculty. For example, during the 2023–2024 academic year, students received training in medical improvisation (Watson 2011; Fessell et al. 2020) in which the instructor adapted improvisational theater principles and training techniques (e.g., experiential exercises, debriefs, and discussion of applications to medicine) to teach medical students how to improve communication, cognition, and teamwork in the field of medicine.

What is learned in the classroom setting is subsequently strengthened with community-based experiences throughout the curriculum. Specifically, medical students learn from community health workers in the rural villages around UGHE’s campus, for instance, by accompanying community health workers on mental health home visits, an educational model that provides them with a profound understanding of the realities of problem-solving in under-resourced settings. UGHE’s community engagement strategy has fostered strong and mutually beneficial partnerships with its host district. Partnerships in the Burera district, along with the Butaro Hospital, provide place-based learning, which emphasizes and embraces community engagement. For its work in this area, UGHE was recognized with an ASPIRE to Excellence Award in place-based health professions education in 2022 from the Association of Medical Education in Europe (AMEE) and ranked eight among 117 sub-Saharan universities for teaching, research, and social impact in 2023 by the Times Higher Education (Times Higher Education 2023).

## Competencies Associated with the Liberal Arts Approach

In the context of UGHE, the investment in liberal arts classes, pedagogy, and culture seeks to confer specific competencies in students to allow them to be more effective physicians, particularly with regard to promoting health equity. Recognizing the lively debates about competency-based medical education in the literature (Eichbaum 2015), UGHE has focused on competencies that allow physicians to take a critical stance on individualist, hierarchical approaches and reflect instead capacities to collaborate, communicate, and work with communities to improve global health equity. UGHE identified five competencies (summarized in Table 1) developed across the curriculum: 1*) oral and written communication* across multiple media (which enables physicians to undertake effective advocacy, leadership, and collaboration at individual and systems levels); 2) *critical and reflective thinking* (which allows physicians to challenge the norms of persistent health inequity and scarcity in the globe); 3) *synthesizing new ideas and multiple perspectives* (which encourages physicians to seek creative, non-obvious approaches to age-old health challenges; 4) *engaging with ambiguity and adapting to dynamic situations* in inclusive and productive ways (which fosters in physicians leadership skills useful in complex health and social systems); and 5) capacity and joy of *lifelong learning* (which opens for physicians a more meaningful life).

**Table 1:** Competencies Related to the Liberal Arts in the Education of Health Professionals

Competency	Social medicine skill	Example courses	Pedagogy
Oral/written communication across multiple media	Advocacy; leadership; collaboration; expressing empathy	Media and health; communication and writing	Original writing and class presentations as individuals and groups
Critical and reflective thinking	Understanding root causes of health disparities; making diagnoses; self-awareness and self-care; pragmatic solidarity	Scientific reasoning, history, psychology, anthropology	Regular journaling, personal and essay writing, group dialogue; original primary research projects; writing and integrating field notes
Synthesizing new ideas and multiple perspectives	Creative problem solving; understanding others; empathy; innovating to promote health and health equity; cultural humility	Multiple across humanities and arts, social science, and natural sciences	Reading multiple sources; taking others’ views in presenting; integrative papers
Engaging ambiguity and adapting to dynamic situations	Leadership; teamwork; community convening and development	Multiple	Individual and group development exercises; debriefs; field visits, community-based, collaborative work
Lifelong learning	Finding meaning; staying relevant; building resilience; mentoring others	Multiple	Primary research; self-designed topics

If a liberal arts approach—including multidisciplinary courses, an inquiry-based pedagogy, and a culture undergirded by intellectual freedom and equity in the classroom and on campus—thrives in medical education, we hope graduates will be better equipped to view clinical problems from multiple perspectives, to practice cultural humility (Farmer 2016; Tervalon and Murray-García 1998), and to work collaboratively with patients and their families. We also hope they will be more likely to conduct research on and ultimately influence the health and social systems in which they work to promote greater health equity. Ideally, they will be ready to lead more inclusively, navigating social and gender power relations in the communities in which they operate and overcoming the risk of burnout as they practice principles of social justice in their work.

The traditional approach to medical education—with a heavy focus on science-based courses, a lecture-based pedagogy that privileges memorization over critical and creative thinking, and a culture based on social hierarchy—has been tried; it is time to consider a different approach. The African continent now houses more than 168 medical schools (Monekosso 2014), and the infrastructure is growing to address severe shortages of health professionals. As the continent is poised to produce many more physicians, it is valuable to pause and think about inspiring a new generation of physicians—who will not be overly narrow purveyors of medical care but rather vital elements of more effective and equitable health and social systems.

## Conclusion

As countries in Africa and around the world work to develop health professionals who understand the social determinants of health and embrace their roles in promoting health equity, including the liberal arts in health professional education can make a difference. The approach—through multidisciplinary classes, innovative pedagogy, and a culture of equity and inclusion—can transform narrowly focused educational degrees and instead create physicians and other health professionals who are prepared to lead in the health and social systems in which medical care is embedded. Several studies have concluded that this educational approach has measurable benefits. The literature is limited by studies with sample sizes and methodological weaknesses; however, the consistency of results is important, and further research is warranted. In particular, qualitative studies that explore the nuanced mechanisms by which such educational approaches enhance competencies, blunt burnout, and result in more effective medical practice are needed. In time, interventional studies with longer-term follow-up will be helpful to understand and spread best practices in education for global health equity.

## Data Availability

Not applicable.

## Data Availability

The data matrixes produced during the current investigation are only accessible from the corresponding author on a justifiable request.
